# A Contribution to the Hereditary and Pathological Aspect of Vice

**Published:** 1883-02

**Authors:** G. Frank Lydston

**Affiliations:** Late Resident Surgeon of the Charity and State Emigration Hospitals, New York. Lecturer on Genito-Urinary and Venereal Diseases at the College of Physicians and Surgeons, Chicago; 137 E. Madison Street


### ARTICLE II.

A CONTRIBUTION TO THE HEREDITARY AND PATHOLOGICAL ASPECT OF VICE. By G. FRANK LYDSTON, M.D., Late Resident Surgeon of the Charity and State Emigration Hospitals, New York. Lecturer on Genito-Urinary and Venereal Diseases at the College of Physicians and Surgeons, Chicago. (Read before the Chicago Pathological Society, December 11, 1882.)

The hereditary and pathological aspect of vice in its protean forms, is a subject which has, of late years, attracted considerable attention at the hands of a few enthusiastic observers, and has been pretty thoroughly and exhaustively analyzed, but it has not, however, received sufficient attention from either medical or civil investigators to render superfluous further discussion of the subject by those who enjoy the relatively rare opportunity of practical observation of our criminal classes. I have, therefore, taken the liberty of presenting the conclusions drawn from somewhat extensive personal observations, although mainly corroborative of statements made by higher authorities upon the same subject.

There seems to be but little doubt that morbid physical conditions are often responsible, indirectly, for many of the phases of conduct that we term criminal. An individual who is suffering from severe pain is not in a condition of stable mental equilibrium, as many persons may be able to verify by introspection under such circumstances, and it is still more evident that one who is subject to profound nervous disorder is likely to suffer from impairment of the higher mental faculties, as evidenced by enfeebled will, and illogical reasoning, associated with a perverted moral sense. And not only the nervous system, *per se*, but through it, indirectly, disturbed functions of the structures of organic life may also be responsible for disturbed mentality. In this connection I will venture to quote a passage from a recent address by Dr. George Perkins, at the opening exercises of the University of Vermont:

"A man whose digestive system is a continual scourge, whose nervous system is feeble or irritable, whose brain receives a supply of impure blood, cannot be the same man morally that he would be were he in the full vigor of health. Well men are not always virtuous. Ill health is not always the sole cause of crime, nor can criminals be treated as invalids; yet crime is often both the parent and offspring of disease. Since we may not know the exact relation of pathological conditions to crime, we must punish crime as such, but because of our ignorance as to its cause, we may be liberal with our charity and lenient in our judgment, whenever, and to such extent, as they may not interfere with the welfare of society as a whole."

The doctor evidently appreciates the fact that more law than justice is dispensed to criminals sometimes, but in the light of our present knowledge, it is difficult to see a solution of the problem which will serve to remedy the evil, or prove satisfactory to society at large.

The leniency and sympathy of the few are not likely to prove of great practical value to the criminal. I do not think that the influence of disturbed organic functions upon the mind is at all over-rated. The association of melancholia with a disordered liver, or of gout and a bad temper, may serve as striking examples of this fact, and hardly any one would claim that cheerfulness and a bad digestion are not incompatible. To determine an anatomical or pathological basis for crime is by no means an easy problem, but it has recently received considerable study from industrious scientists, although their results have been by no means conclusive. The studies of Benedikt and Osler, in this direction, have been especially interesting. Benedikt claims that in criminals the brain is atypical, and different from the average human brain in the cultured races. The variations consist chiefly in the atypical conformation of certain gyri and fissures, there being a confluence of many primary fissures, and there being found four horizontal convolutions. The experiments of Osler are, to a certain degree, confirmatory of Benedikt's, although not quite so definite. He claims, however, that the confluent fissure type is not so prominent as stated by Benedikt. A certain amount of disparity in the results of the two observers may be ascribed to the probable fact that the American criminal type is not so strongly marked as the European. The chief sources of fallacy are, that this atypical arrangement of the fissures and gyri may co-exist with a superior general development of the convolutions, and that it has not yet been proven that the same arrangement does not exist in other classes of individuals. Further experiment and observation will be necessary to determine the co-relation between certain mental attributes and the confluent fissures and increased number of frontal gyri. The experiments of these observers have been solely with reference to the criminal cerebral type, but observations upon the condition of other organs in habitual criminals have been made by others. The results of the observations of Bruce Thompson, in the general prison of Scotland, are striking. To use his own words: "In all my experience, I have never seen such an accumulation of morbid appearances as I witness in the post-mortem examinations of the prisoners who die here. Scarcely one of them can be said to die of one disease, for almost every organ of the body is more or less diseased." These results do not differ much from those seen post-mortem in almost any large public hospital in which a large number of cases from the lower classes are treated, so that they can hardly be adduced as evidence of the pathological nature of crime. Most of the morbid changes which are responsible for many criminal actions are not physically demonstrable, but are too occult to be detected by any means of investigation which are, or perhaps ever will be, at our command. These changes are mainly in the nervous system, and oftentimes give manifest morbid results, even when the exact condition is not discernible. Another point to which I would call attention is the statement of Thomson, to the effect that physical defects and deformities were remarkably frequent among the convicts under his charge. My own observations do not confirm this, inasmuch as the physique of the men whom I observed was fair, on the average, and the proportion of physical deformities no greater than might be expected in any community of a similar size, although those that did exist were frequently very marked.

My own experience with the American criminal as represented by those confined in the Blackwell's Island Penitentiary, and which may be taken as a type, differs from that of Thomson in some respects. I found that the men were of fairly robust physique, on the average, and that the proportion of men who were really ill, aside from venereal and alcoholic affections, was comparatively small for such a large community, living under circumstances so favorable for the development of disease.

Malingerers were, of course, numerous, and their pertinacity, and morbid appetite for specially prepared mixtures of green soap and assafœtida was certainly surprising; but the percentage of genuine invalids was quite small. Those cases of sickness which did occur, however, were often due to profound visceral lesions, but could generally be traced to the patient's habits of life, intemperance and exposure, with, in some cases an organization hereditarily defective. The reason for this difference in the results obtained by myself and those claimed by Thomson, is partially explicable from several considerations. The most important of these is the fact that the majority of metropolitan criminals, when so affected by disease that they can no longer carry on their avocation, which requires a considerable amount of physical vigor in its pursuit, enter the various charitable institutions and there terminate a miserable existence.

I found, therefore, in the Charity Hospital, the patients of which were recruited to a large extent from the very slums of the city, almost as great a variety and extent of pathological changes as those described by Thomson. A certain proportion of these patients must necessarily be excluded from the criminal type proper, thus diminishing the relative proportion of pathological changes found in the latter. There is no question in my own mind, but that the average results obtained by observations upon European and American criminals differ considerably, for another quite important reason besides that just given, and to which I will hereafter call attention.

The preponderance of the neuroses among the affections to which the criminal classes are subject, is strongly insisted upon by Maudsley, and in my own experience, this has been fully corroborated, for although, as I have before stated, the majority of the convicts were healthy and comparatively robust, there was a strongly marked neurotic element in a large proportion of the cases of sickness requiring medical attention; epilepsy and neuralgias, and among the women cases of hysteria, being especially frequent. Cases of true insanity, however, were relatively infrequent, and I doubt whether many communities of a corresponding size, could show as few of the mentally disordered, as could that body of convicts.

Much has been said of the mental inferiority of the average criminal, and I have myself found that, as a class, they are mentally defective, often to the extent of imbecility, although often possessed of an extraordinary amount of brutish cunning. Occasionally, however, I have seen a really bright intellect among them, but generally in sporadic instances of criminality from the better class of the community, in whom necessity or acquired propensities were to a great degree responsible for their actions. The ordinary criminals appear to form one great genus.

Thomson states that he has never seen any manifestations of genius or artistic taste among criminals. On the contrary, there was seldom any difficulty in finding men of decided talent in the hospital under my charge. Exceptionally clever workmen in wood, such as expert cabinet-makers, experts in the manufacture of fancy articles and in carving, and skillful penmen, were nearly always at hand, even in cases in which the moral sense was extremely low. Probably the most important question relative to criminals is that of their moral status.

The assertion is often made that a certain class of our criminals are morally insane, and I think there is hardly a question of its truth. In nearly every instance of crime among habitual criminals, the question of the morality of the action is never considered, and indeed, there seems to be an entire incapacity for its appreciation. With these beings, crime is regarded as a business, subject to certain disadvantages, but having somewhat of the fascination of a game of chance. Their ideas of right and wrong seem to be based solely upon the extent to which their personal interests are involved, and the amount of risk of punishment incurred in the performance of a criminal action to attain certain ends. Their ideas of morality are not those engendered by an instinctive sense of right and wrong, but to them it is something arbitrarily fixed by social custom for the protection of society, their natural enemy, a conformity to which is enforced by law, and a violation of which renders them liable to punishment. This absence of the moral sense is not confined to those criminals whose mental powers are of a low, or almost imbecile type, but it is also to be found among the more intelligent of them. When we consider this moral defect in the mental status of the more intellectual type of criminals, we can much more readily explain the failure of all moral persuasion in the case of the lower orders, in whom the mental state borders closely upon imbecility. To quote Maudsley, "Impulses to theft, incendiarism and violence are quite frequent in cases of imbecility, in which the passions are strong, and the intelligence feeble." We may readily appreciate a condition of moral imbecility, if we are at all observant of the creatures who fill our police courts and jails.

There is one peculiar fact which I have often noticed with reference to the moral sensibility of criminals. A criminal may be utterly oblivious to all moral considerations in many directions, and yet have a species of contempt, or even abhorrence, for certain phases of vice. Strange as it man seem, there is a certain degree of caste even in a prison, and the supreme contempt evinced by some of the assault and battery men for their brethren of the larceny list, among the criminals under my charge, was amusing in the extreme. In this connection, I will cite the case of the Rev. Brother Cowley, who, it will be remembered, served a time in the New York penitentiary, for his maltreatment of young children who had been consigned to his care in the so-called "Shepherd's Fold." I have seldom seen a person held in such aversion and contempt as was this man by the rank and file of the prison. The appellations bestowed upon him were more expressive than elegant, and I remember numerous arguments between the other convicts and himself, in which he endeavored to try the effect of moral persuasion upon them, and in which the reverend rascal got decidedly worsted. These men, ordinarily so completely lost to all moral impressions, and who deemed highway robbery and murder incidental to their lives, and the most natural thing in the world, although subject to the risk of detection and punishment, had sufficient manhood to loathe such inhumanity as had been practiced by this educated fiend.

Analogous instances are numerous but I will cite but one other example. In the female department of the penitentiary hospital were criminals of all grades, from the accomplished female confidence operator, down to the petty larceny and shop-lifting class, and there was ample opportunity to observe the peculiarities of female criminals. There was as great a degree of caste among them as could be well imagined, and among the better class, the lower orders of viciousness and wrong-doing were held in great aversion. Perhaps the most striking instance was that of Madame Berger, the notorious abortionist, who was under a five years sentence for causing the death of a well-known young woman of Brooklyn, a case that created considerable excitement at the time. This woman was held in about the same loathing, even among some of the lowest of these female dregs of society, as was Brother Cowley among the men. She was avoided and shunned by nearly all of them.

I have spoken of the neuroses as affections which have been strongly insisted on as a basis for crime, and have also stated that the neurotic type, in my experience, prevails among the disorders to which criminals are subject, and in reference to this point the question may be asked, whether the sequence of cause and effect is at all definite? and whether the same tendency to crime would not exist if these affections or neuroses were not present?

It must be confessed that the problem is by no means clearly solved, and we must acknowledge the fact that crime is frequent in cases in which there appears to be no neurosis or other predisposing cause. I think, however, that crime would not be by any means so frequent, were these causal elements eliminated. They undoubtedly lessen the power of volition and self-control, in the same manner, if not in the same degree, that they do in the higher walks of life, in which criminal tendencies are relatively infrequent, but in which the predisposition probably exists in many instances, and crime would actually occur, were the circumstances and necessities of the individual such as to act as existing causes. It may be argued, that there being a defective nervous organization among the lower types of humanity, as represented by the criminal classes, we might conclude, *a priori*, that actual structural disease, or even functional disorder, would in them act as a more powerful causal element of crime than in persons in the upper strata of society, in whom the nervous organization is more perfect, and the mental discipline better. This argument is a valid one, but is, I think, counterbalanced by the disadvantages accruing from that same greater perfection of organization, which is readily appreciated, when we consider its greater sensibility and more powerful tendencies to emotional and erratic impulses, which are such prominent factors in the neuroses among our better classes.

In the lower ranks of humanity, a defective cerebral development is the rule, and it is from this lower class that the criminal element of society is chiefly derived. Now, it would be difficult to determine precisely how great an influence the causal elements of necessity and privation exert in such people, but it is obvious that there is something more than these factors required to explain the prevalence of crime among them; and what could be a more logical explanation than the fact of the existence of a defective cerebral development, with a consequent inferiority of the higher faculties of the brain, and a predominance of animality, which is so evident in the majority of criminals? A certain proportion of these persons might be saved from criminal impulses by proper training and discipline, and removal from the baneful influences by which they are ordinarily surrounded, but by far the larger number of them are afflicted with a nervous organization primarily so defective, that no amount of pains would serve to prevent the development of criminal tendencies on the occurrence of the slightest exciting cause. In this connection, however, we are compelled to acknowledge the well-known fact that the difference in the relative frequency of crime in the two extremes of society is, to a great extent, due to the more powerful influence of temptation and necessity in the one than in the other. This does not at all refute our argument as to the existence of defective mentality as a cause for crime in the lower walks of life, for, as we have seen, a certain proportion of the lower class of criminals might be saved by proper training and influence, while a certain proportion of individuals of the higher classes are possessed of an inherent predisposition to vice, which is liable to develop into actual crime upon the occurrence of any exciting cause.

Acquired tendencies probably have much to do with the causation of crime in many cases, and this statement holds good especially in the case of the American criminal class, in whom the local effects of the co-operative influences of time, heredity and natural selection, in each community of low-bred criminals and wrong-doers, are not so manifest as in Europe. The same explanation would suffice for the disparity of results obtained by Benedikt and Osler, and for the statements of Thomson as to the frequency and multiformity of pathological changes, and the low brutishness noted in the criminals found in the Scottish prisons. In summing up the evidence in favor of a physical basis for vice, I think that a defective or imperfect general cerebral development, with its attendant predominance of the animal type, or superior development of the lower brain centers, must be conceded to be a powerful cause, and although the co-relation of actual pathological changes and crime is not at present demonstrable, or at all definite, the powerful influence of bodily ailments upon the higher functions of the brain must be alowed by every one. The old adage of "*mens sana in corpore sano*," is perhaps nowhere more *apropos* than in the consideration of the causes of many instances of criminality.

I have spoken of the influence of heredity, which is so strongly set forth as a cause of crime, but only with reference to the hereditary transmission of physical defects and pathological conditions which react upon the mental and moral status of the individual. In addition to this influence of heredity, there is a special influence that is more important, and which is by no means a new consideration, having been recognized by most of the authorities who have given their attention to this subject. I refer to the hereditary transmission of intellectual and moral peculiarities, which as truly follows a definite law as does that of physiological and pathological conditions. An hereditary tendency to vice undoubtedly exists, and must be recognized in certain instances, as fully as are hereditary tendencies to disease of special types. The imprint of heredity npon mentality may be quite as strongly marked as it is upon the animal organization itself, and the exercise of the mental powers in any vicious direction is as surely transmitted to future generations, in many instances, as a predisposition to vice, as is a taste for art, or scientific pursuits, in beings of superior intellect. To quote the words of Maudsley, "Just as a man may inherit the stamp of the bodily features and characters of his parents, so he may also inherit the impress of their evil passions and propensities. Of the true thief, as of the true poet, it may be said, that he is born, not made." Although this position is, in many instances, supported by fact, yet there are many cases in which it would be difficult to exclude the influence of circumstances and acquired tendencies. Ribot, in his excellent work upon heredity, touches upon the hereditary nature of vice. He makes the statement that such evil propensities as avariciousness, drunkenness, dishonesty, thieving, and a passion for gaming, are very often hereditary, and as an illustration of the well-known influence of heredity in the production of thieving propensities, he cites instances where whole families were addicted to stealing and like offenses. He also cites the case of a lady, who was afflicted with an inveterate passion for gambling, and whose son became a most reckless profligate and gambler, his evil tendencies developing when he was not at all subjected to the baneful influence of his mother's example. Whether t is instance resulted as a consequence of the mother gambling during the period of her pregnancy, is not stated, but some might take that view of its causation.

It is a demonstrable fact that children of illegitimate parentage are peculiarly liable to become addicted to vice and crime, as a result of an hereditarily deficient moral sense, although it cannot be denied that such children are not usually favored with the tender care and judicious training bestowed upon children born under more favorable auspices, and that this has much to do with the development of immorality. Even when they are placed under the most favorable circumstances, however, their hereditarily vicious propensities are liable to exhibit themselves from the action of exciting causes which are many times remarkably slight. Consanguinity in the parents has doubtless as powerful an effect upon the moral and intellectual status of their offspring, as it often has upon the physical, which is so well recognized, and moral imbecility, or, at least, a perversion of the moral sense, in the children, is not unlikely to result from such unions. Its effect in producing ordinary mental inferiority, or even insanity, is fully recognized. Parents possessing an unstable nervous organization, and who are perhaps afflicted with neuralgias of various kinds, epilepsy, chorea, hysteria, or allied affections, are also usually of inferior will power, and are not logical thinkers. They are quite likely to be erratic and notional, or a "little odd," as it is often termed. These defects are frequently transmitted to their offspring, and perhaps are intensified in them, and but too often associated with defective mentality, and a certain degree of moral anæsthesia.

We have, then, a strong predisposition, if not an inherent tendency to crime, and it is obvious that when it occurs, there are several factors in its production which should be recognized. These are, first, a nervous organization primarily defective, and either in a condition of actual disease, or strongly predisposed thereto. Second, a defective moral sense, or, so to speak, a moral anæsthesia. Third, a faulty and illogical reasoning power, which is liable to subvert the interests of the public to the seeming welfare or pleasure of self. Fourth, a vacillating and feeble volitional power. Sometimes, too, we have superadded to these causal elements, that of actual organic disease, with its attendant exaggerations of the moral perversion already existing. Against these powerful influences, especially when, as is often the case, they are associated with improper training or example, and the forces of necessity and temptation, many of the poor creatures so afflicted are unable to contend. The same argument is applicable to those unstable nervous organizations which so often become the victims of dipsomania. It is a well established and almost indisputable fact, that there are few more powerful factors in the production of drunkenness than heredity. It is not my purpose to enter upon the various causal elements of inebriety, but merely to mention this single predisposing cause, which is perhaps one of the most important to be considered. Here, again, we have a faulty nervous structure, the equilibrium of which is easily overthrown, in addition to a will power hereditarily weak. It is also true that traits of character, and nervous and mental defects which are acquired in one generation, may be transmitted to the next by the inexorable power of heredity, and so on for successive generations, retrogression being enhanced by acquired proclivities and moral conditions in each, until finally the race or family becomes extinct, nature thus relieving society of these moral pests.

There is something to be said with reference to the influence of uncontrollable impulses in the production of crime, and it must be conceded that deeds of violence such as murder or assault, often result from what must be termed "an uncontrollable impulse." But impulses to do violence under certain provocation, perhaps, arise in the best of us at times, and why is it that one should have the faculty of self-control rather than another? There may be no deficiency of the moral sense, *per se*, in an individual, and yet under proper stimulus he may commit murder. Have we not here, also, in many cases, the imprint of heredity in the form of defective mentality, with a consequent deficiency of volitional self-control as a predisposing cause to the act of violence? Very few murderers entertain the slightest idea of the moral relations of the act at the time of its commission, or reflect upon its consequences, but as the result of some great excitement from real or fancied wrongs, or the effect of alcohol in a subject in whom the power of self-control is hereditarily deficient, the small amount of that faculty he possesses is lost for the time, thus giving his evil animal passions full sway, and leading to a deed of violence, which, aside from the dread of consequent punishment, he may forever afterward regret. Many times, however, a murderer may regard his deed of violence as an eminently proper thing to be done, and as the only measure which will, in the least, satisfy his thirst for revenge or reparation, and indeed there are some injuries that cannot be repaired in any way, nor can anything but the life of the offender even approximately atone for the wrong inflicted. The self-consciousness of having violently deprived the offender of life, is a measure of satisfaction to such homicides. The man who kills the destroyer of his domestic happiness, or the betrayer of his sister, may be momentarily insane at the time of the discovery of his wrongs, and may kill the offender while such insanity exists, but he may, on the other hand, be mentally and morally sound, and regard the case, not from its legal aspect, but from the standpoint of justice. Could any moral persuasion, however strong, convince such a man of the immorality of the act which he meditates? Not but that in after years he may regret the deed, however justly done, for I doubt much if there are many men, not morally defective, who can slay another, even in self-defense, without having an impress made upon their lives and characters which will tinge them ever after. I make this statement as a matter of personal observation. The fear of punishment often restrains a man from homicidal impulses, even when he is convinced of the justice of his cause, and would follow the old Mosaic law to the letter, if allowed to follow his own judgment and inclinations. Cases of this kind must often be excluded from our list of criminals who are predisposed to acts of violence by a defective mental and moral tone, but they may, on the other hand, be just such cases as we have considered, in which the injury suffered constitutes a more than sufficient exciting cause for the complete overthrow of all moral reasoning, and a thorough subjugation of the higher faculties of the mind to the merely animal impulses.

A very important phase of vice, and one which merits attention with reference to the influence which heredity exerts in its production, is prostitution. This is a subject which has received pretty thorough ventilation at the hands of enthusiastic moralists who have been earnestly seeking for a means of correcting or regulating the social evil, but with little success.

These people, it seems to me, take a very one-sided view of the matter, and make very little allowance for certain elements in the production of this vice, which I believe are so important. Some ascribe their want of success in controlling the evil to the fact that "the supply is regulated by the demand," and arguments of that nature. Now there is doubtless much truth in this assertion, but would it not be well for them to investigate the matter a little more thoroughly, and in a more liberal manner, with a view to the discovery of more logical and powerful causes? The same hereditary influences are brought to bear in the production of prostitution that we have found to exist in the causation of the various forms of vice, and immorality in general. We have the same congenitally defective nervous organization, and the same moral insensibility that we have seen in connection with other vicious tendencies, or we have a similar transmission of mental and moral peculiarities; and it is my opinion that a large proportion of the women who are leading lives of shame are indebted therefor to an innate tendency to immorality, due to a deficient appreciation of the moral law, which, under favorable auspices, becomes a permanent factor of the daily life of the individual. This inherent immoral proclivity may not be hereditary in all instances, but may be peculiar to the individual, there being an immediate family line noted for virtue and uprightness, as is frequently the case, and it matters not whether it consists in a predominance of the animal passions alone, the reason and moral sense being unimpaired, but temporarily overpowered, or in a diminished appreciation of the moral law, associated with a feeble volitional power, the innate predisposition remains the same, and in either case may be hereditary. The predisposition existing, the exciting cause is of merely secondary importance, and such influences as the love of admiration and dress will be found to be the causes of fully as many instances of falls from grace, or at least of the adoption of a life of shame, as are the temptations and deceptions spread for the unwary by unprincipled persons.

Slight causes, indeed, are a fondness for admiration and dress, when contrasted with the moral law and other influences which are brought to bear upon a woman, with a view to keeping her in the right path, and only a recognition of the existence of a deficient appreciation of that moral law, will render such cases explicable. An admirable instance of the effect of such influences as I have given is cited by Sanger, in his history of prostitution: A family of seven sisters were induced to leave a moderately comfortable home, in a New England town, and enter a life of shame in the metropolis, one after the other, the sole inducement being the life of luxury and ease which such a course was supposed to bring. They happened to establish themselves in the same locality, occupying a number of buildings upon a prominent street, which has since borne the appellation of "The

Sister's Row." This is by no means an isolated instance, for it is a notorious fact that the proprietors of houses of ill-repute in New York City make, at more or less regular intervals, visits to the smaller New England towns and villages, for the purpose of recruiting their supply of boarders, and it is also true that there is usually a more or less definite understanding as to the life which they are expected to lead, although painted in glowing colors as to its luxuriousness. Surely, there must be a defective moral sense in such cases, for no matter how pleasant the prospective life, as depicted by the wily procuress, the fact that it is a life of sin could not be otherwise overlooked, or, at least, ignored.

Necessity, it is true, is at the bottom of many cases, but even this is many times more apparent than real, for how very many of these people entertain the most extravagant notions as to their necessities, and would not be satisfied with a life devoid of luxuries. Then, too, the predisposition existing, an excuse so valid as that of necessity, is very acceptable to one whose moral sensibility is already of a low grade, and I think that this moral anæsthesia is really the primal source of many of the departures from virtue and adoption of a life of vice. With reference to those who are really unfortunate, of whom there are but too many, I think that they comparatively rarely enter a life of shame, but seek to conceal their misfortune, and are but too anxious to redeem themselves should the opportunity offer. In spite of the views of many one-sided moralists upon this matter, I think that it will be found that in nine cases out of ten of the upper classes of the demi-monde, the life of the woman is one of choice rather than compulsion, and that the majority of them would not alter their mode of life if they could, and I have seen the experiment of redemption tried too often to believe otherwise. Exceptionally, through religious enthusiasm, a reformation may occur, but many of such converts are very prone to backslide. Those of the women of ill-repute who signify their willingness to adopt an honest life, are usually those whose lives have become squalid and miserable, and who are, perhaps, afflicted with disease. These poor creatures are, of course, but too willing to leave a life which no longer has charms for them, and which holds out no other prospect than that of necessity, and a miserable death. While their attractiveness yet exists, and they are in demand, they can command a life of luxury and ease, and moral persuasion has but little effect upon them. Many cases of prostitution arise among the typical lower criminal classes, the same influences being brought to bear as affect the production of other vicious tendencies among such people. These cases are usually those in which there exists a sufficient amount of personal attractiveness to render a life of shame more profitable than one of petty thieving. Such women eventually drop back into the lower strata of criminal life when the period of their usefulness in their more gilded existence has departed. In such cases the innate tendency of their organizations inevitably causes them to gravitate lower and lower in the scale until they reach the very depths of degradation. Anyone who has observed at all closely the class of beings found in our houses of correction or charitable hospitals, particularly those in which a large number of cases of venereal diseases are treated, will be able to attest the truth of this. I have said but little in regard to the pathological aspect of prostitution or to the effect of acquired tendencies, but these are phases of the subject which undeubtedly exist, and which must be recognized.

It is necessary to say but little upon either of these topics however. The influence of the neuroses in the production of prostitution is quite as manifest as in the case of other vicious proclivities, and exerts itself in much the same manner, causing the same moral perversion in this as in other directions. The reflex derangements of the nervous system due to either functional or structural disease of the sexual organs, and attended by a deranged sexuality with its coexistent moral perversion, constitute a probable cause in some cases, as will be evident when the exaggerated results of these disturbances in the form of erotomania and such conditions are taken into consideration. With reference to acquired tendencies, it is obvious that unless proper teaching and example be brought to bear, and evil influences iliminated, a departure from the right path is quite liable to occur; and how much more potent evil influences are likely to be, if, as in the cıse of illegitimate children who are primarily the offsprings of evil passions, a strong principle of heredity exists as a predisposing cause. The most prominent question arising from the considerations which I have endeavored to present in this paper, is that of the proper remedy for the prevention or diminution of the various phases of vice, as deduced from the causes set forth. It must be confessed, however, that in this respect we are not very clear, and it is not at all likely that we will ever attain any very marked results in this direction until the co-relation of cause and effect is much more definitely settled than it is at present.

It is evident that if the general bodily health has a direct influence upon the mind, the medical man can, and probably does accomplish quite as much indirectly in improving the moral status of society as can the church, or, what is often times more efficacious, that wholesome and definite dread of legal punishment which is at present the chief protection of society. Education and proper mental and moral discipline, with a removal from temptation and other malign influences, will save quite a number of individuals, even if possessed of a certain amount of inherent predisposition. Like a predisposition to disease, a predisposition to crime need not necessarily develop into actual lawlessness if proper steps are taken to prevent it.

It does not necessarily follow from the statements embraced in our consideration of the causes of crime, that punishment may not be efficacious in its prevention, even where morbid physical conditions are responsible for the evil tendencies of the individual, for even insane persons, as claimed by Maudsley, may restrain their morbid impulses where they have such a powerful incentive for so doing as the dread of commitment to an asylum. Much may be done in the right direction by the prevention of marriage between neurotic subjects, and of persons nearly related, and this is as true with relation to the prevention of crime as of insanity.

But no matter how energetic our efforts in these various directions may be, we can but hope to diminish the frequency of crime to a slight extent, and can never hope to control it entirely as long as our knowledge of the co-relation of its causes and effects is as imperfect as it is at present. We will have to accept the inevitable, and look upon vice as we are compelled to regard certain diseases as due to causes more or less definite, but which are as yet only to a slight degree, if at all, under our control. We can, however, as suggested by the address which I have quoted in the early part of this paper, be rather more liberal with our charity and more lenient in our judgment, than if these causes were entirely unappreciated.

137 E. Madison Street.

